# Can Individuals with Suboptimal Antibody Responses to Conventional Antiviral Vaccines Acquire Adequate Antibodies from SARS-CoV-2 mRNA Vaccination?

**DOI:** 10.3390/v14050956

**Published:** 2022-05-03

**Authors:** Wataru Ogura, Kouki Ohtsuka, Sachiko Matsuura, Takahiro Okuyama, Satsuki Matsushima, Satoko Yamasaki, Hiroyuki Miyagi, Kumiko Sekiguchi, Hiroaki Ohnishi, Takashi Watanabe

**Affiliations:** 1 Department of Clinical Laboratory, Kyorin University Hospital, Mitaka, Tokyo 181-8611, Japan; kytrans@ks.kyorin-u.ac.jp (W.O.); s-matsuura@ks.kyorin-u.ac.jp (S.M.); oku_8503@ks.kyorin-u.ac.jp (T.O.); miyagi@ks.kyorin-u.ac.jp (H.M.); kssekiguchi@ks.kyorin-u.ac.jp (K.S.); 2 Department of Laboratory Medicine, Kyorin University School of Medicine, Mitaka, Tokyo 181-8611, Japan; satsuk@ks.kyorin-u.ac.jp (S.M.); sugarchild.mt@gmail.com (S.Y.); onishi@ks.kyorin-u.ac.jp (H.O.); twatanab@ks.kyorin-u.ac.jp (T.W.)

**Keywords:** coronavirus disease 2019, severe acute respiratory syndrome coronavirus 2, mRNA vaccine, anti-spike antibody, neutralizing antibody

## Abstract

In Japan, healthcare workers (HCWs) are vaccinated against measles, rubella, chickenpox, mumps, and hepatitis B to prevent nosocomial infection; however, some do not produce sufficient antibodies (“suboptimal responders”). This study compared immune responses to a severe acute respiratory syndrome coronavirus 2 (SARS-CoV-2 mRNA) vaccine among HCWs with normal and suboptimal responses to conventional vaccines. In this prospective cohort study, 50 HCWs received two doses of BNT162b2 mRNA vaccine 3 weeks apart. SARS-CoV-2 anti-spike antibodies were measured 11 times, starting before the first vaccination and ending 5 months after the second vaccination. Antibody titers of four suboptimal and 46 normal responders were compared. SARS-CoV-2 neutralizing antibody activity was measured twice in suboptimal responders, 1 week/1 month and 5 months after the second vaccination. The SARS-CoV-2 anti-spike antibody was detectable in the samples from suboptimal and normal responders at each timepoint after vaccination. Suboptimal responders exhibited SARS-CoV-2 neutralizing antibody activity 1 week/1 month as well as 5 months after the second vaccination; however, activity was slightly reduced at 5 months. Our findings show that suboptimal responders do acquire adequate SARS-CoV-2 anti-spike and SARS-CoV-2 neutralizing antibodies from vaccination to prevent SARS-CoV-2. SARS-CoV-2 mRNA vaccines should thus be recommended for both normal and suboptimal responders to conventional vaccines.

## 1. Introduction

Coronavirus disease (COVID-19), which is caused by severe acute respiratory syndrome coronavirus 2 (SARS-CoV-2), has been declared a pandemic [1,2,3,4]. Vaccination against SARS-CoV-2 infection is important for preventing severe illness and death due to COVID-19.

SARS-CoV-2 has an enveloped, single, positive-stranded RNA genome that encodes four major viral structural proteins, namely, the nucleocapsid, spike, envelope, and membrane proteins; the latter three proteins are found in its membrane. The spike protein guides viral entry into host cells by binding to angiotensin-converting enzyme 2 (ACE2) receptors, which are widely expressed on epithelial cells and macrophages [5,6,7] and is thus an ideal target for messenger RNA (mRNA) vaccine development.

The BNT162b2 mRNA vaccine (Pfizer–BioNTech) is highly effective at preventing clinically significant COVID-19 [8,9,10,11], reducing the incidence of asymptomatic infection and associated infectivity [12,13], and preventing the spread of COVID-19 [9]. The BNT162b2 vaccine was the first SARS-CoV-2 vaccine to be approved in Japan, and priority vaccination of healthcare workers (HCWs) began in February 2021 in order to safeguard the medical delivery system [14].

HCWs also have a high risk of infection with other contagious viruses such as measles, rubella, chickenpox, mumps, and hepatitis B. Therefore, in Japan, HCWs are vaccinated against these viruses to prevent nosocomial infections, and viral antibody titers are measured to ensure the presence of protective antibody levels [15]. However, some individuals do not develop adequate antibody titers against contagious viruses even after vaccination. Such individuals are called suboptimal responders [16]. It is unclear whether suboptimal responders to conventional vaccines can produce adequate SARS-CoV-2 antibodies, including neutralizing antibodies, after vaccination with the BNT162b2 vaccine to protect them against SARS-CoV-2 infection. Therefore, in this study, we investigated whether suboptimal responders acquire SARS-CoV-2 anti-spike and SARS-CoV-2 neutralizing antibodies after vaccination with the BNT162b2 vaccine.

## 2. Materials and Methods

### 2.1. Participants

This prospective cohort study was conducted at Kyorin University Hospital in Tokyo, Japan. Fifty HCWs received two doses of the BNT162b2 vaccine 3 weeks apart. The participants were HCWs working at the hospital who agreed to participate in the study and to periodically donate blood samples before and after vaccination. Those whose antibody levels were below the standard values [17] for preventing infection after receiving two or more doses of vaccine against measles, rubella, chickenpox, mumps, or hepatitis B viruses were classified as suboptimal responders.

### 2.2. SARS-CoV-2 Anti-Spike Antibody Assay

SARS-CoV-2 anti-spike antibody measurements were performed on serum obtained from centrifuged peripheral blood using the Elecsys Anti-SARS-CoV-2 S RUO (Roche Diagnostics, Rotkreuz, Switzerland) on a Cobas 6000 (Roche Diagnostics) analyzer via the double-antigen sandwich enzyme-linked immunoassay method [18]. Samples were collected before the first dose of the BNT162b2 vaccine was administered; 3 days after the first vaccination; 1, 2, and 3 weeks after the first vaccination; 1 week after the second vaccination; and 1, 2, 3, 4, and 5 months after the second vaccination. The measurement range of the antibody assay was 0.4–250, and samples with an antibody titer of ≥250 U/mL were assayed again after dilution.

### 2.3. SARS-CoV-2 Neutralizing Antibody Assay

Using serum obtained from the peripheral blood of suboptimal responders after vaccination, SARS-CoV-2 neutralizing antibody activity was measured at Osaka University Microbial Disease Research Association Laboratory. A 50% inhibitory dilution factor (ID_50_) using pseudovirus [19] was measured. ID_50_ values of ≥50 were considered positive [20]. For the neutralization assay, 60 μL of pseudovirus, which is equivalent to 2.5 × 10^6^ relative light units (RLU)/mL, was incubated with 60 μL of serial dilutions of serum samples for 1 h at 37 °C. After incubation, 100 μL of the mixture was added to the Vero cells on a 96-well plate. The cells were lysed 24 h post-infection, luciferase was activated using the Luciferase Assay System (E1501, Promega, WI, USA), and RLU was measured using a Synergy LX analyzer (BioTek, Winooski, VT, USA). Percent neutralization was calculated using GraphPad Prism 8.0.2 (GraphPad Software, San Diego, CA, USA). Percentages of RLU reduction (inhibition rate) were calculated as follows:

1 − (RLU of sample sera − pseudovirus only wells)/(RLU from medium only wells − pseudovirus only wells) × 100%

The titers of neutralizing sera were calculated as ID_50_. The transition of neutralizing antibody activity values after vaccination in suboptimal responders was assessed 1 week/1 month and 5 months after receiving a second dose of the BNT162b2 vaccine.

### 2.4. Statistical Analysis

Nonparametric continuous data are reported as the median and interquartile range (IQR). The Mann–Whitney U test was performed to assess whether differences between groups were statistically significant. Two-sided *p*-values of <0.05 were considered statistically significant. The Wilcoxon matched-pairs signed rank test was performed to assess whether differences in antibody levels within an individual at different time points were statistically significant. To compare the age (ratio of the number of individuals aged 20–40 years and those aged 50–60 years) and sex compositions of the suboptimal and normal responders, Fisher’s exact test was performed to test for statistical significance. The Mann–Whitney U test was performed to compare antibody levels between suboptimal and normal responders 1 week after the second vaccination. All statistical analyses were performed with EZR (Saitama Medical Center, Jichi Medical University, Saitama, Japan), a graphical user interface for R (The R Foundation for Statistical Computing, Vienna, Austria) [21]. Specifically, EZR is a modified version of R commander (version 1.54) that has been designed to add statistical functions frequently used in biostatistics.

## 3. Results

The suboptimal responders comprised three males (L1, L2, L4) with negative (<16.0) anti-measles immunoglobulin G (IgG) antibody titers after measles virus vaccination and one female (L3) with a negative (<4.0) anti-mumps IgG antibody titer after mumps virus vaccination. Forty-six participants, 12 males, and 34 females, had antiviral antibody titers after vaccination against other viruses above the standard value (normal responders). The four suboptimal responders were aged 20–40 years. Normal responders were older than suboptimal responders (57% vs. 0% were aged 50–60 years, respectively), but no significant difference was observed (*p* > 0.99). In addition, the male/female ratio was higher in suboptimal than in normal responders (74% vs. 15%), but no significant difference was observed (*p* > 0.99). All suboptimal responders were non-smokers, none were obese, and none had a history of immunosuppressive drug use or a medical condition associated with immunodeficiency.

### 3.1. SARS-CoV-2 Anti-Spike Antibody Levels

**Figure 1:**
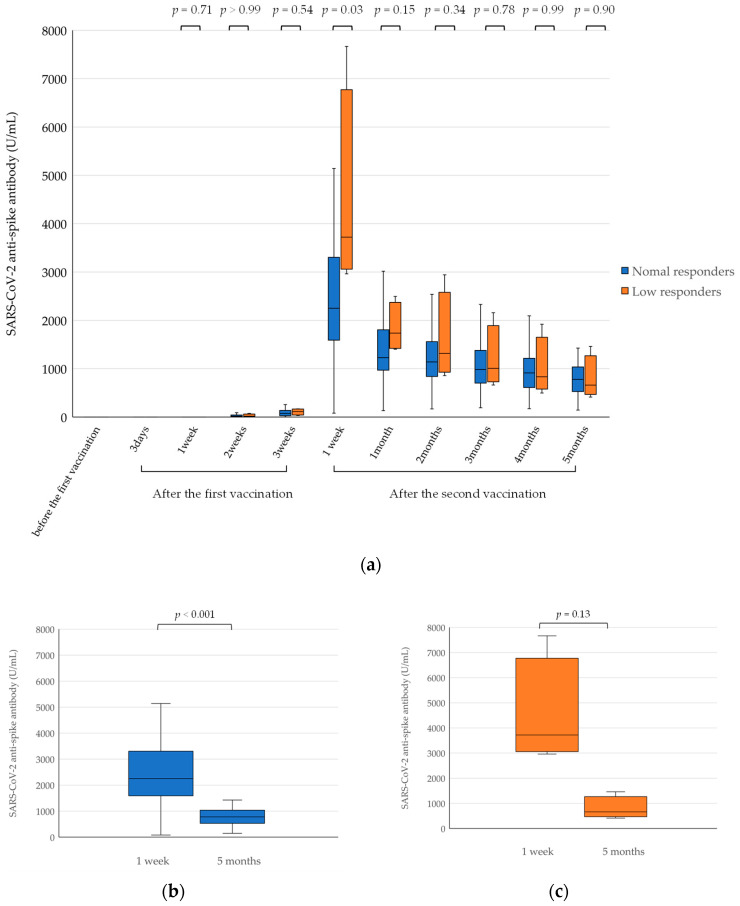
SARS-CoV-2 anti-spike antibody responses in suboptimal/normal responders before and after vaccination with the BNT162b2 vaccine. (**a**) SARS-CoV-2 anti-spike antibody responses over the whole study period (from before the first dose to 5 months after the second dose of the vaccine). (**b**) SARS-CoV-2 anti-spike antibody titers in normal responders 1 week and 5 months after receiving a second dose of the BNT162b2 vaccine. The antibody titers were significantly lower at 5 months than at 1 week after the second dose (*p* < 0.001). (**c**) SARS-CoV-2 anti-spike antibody titers in suboptimal responders 1 week and 5 months after receiving a second dose of the BNT162b2 vaccine. Antibody titers decreased, but the decrease was not statistically significant (*p* = 0.13). The box plot comparisons were performed using the Mann–Whitney U test; *p*-values < 0.05 were considered statistically significant. Suboptimal responders showed low-level antibody responses to previous measles or mumps vaccines, while normal responders showed normal antibody responses to previously received antiviral vaccines.

The median (IQR) SARS-CoV-2 anti-spike antibody titer was 2251.5 (1639.0–3276.3) in the normal responders and 3721.0 (3255.0–4983.3) in the suboptimal responders 1 week after the second dose of BNT162b2 vaccine. This difference was statistically significant (*p* = 0.03) (Figure 1a). One week after the second vaccination, there was no significant difference in total antibody levels between participants aged 50–60 years and those aged 20–40 years (*p* = 0.66).

The median (IQR) SARS-CoV-2 anti-spike antibody titer in normal responders was 779.3 (534.6–1024.3) 5 months after a second dose of the BNT162b2 vaccine (Figure 1b). In suboptimal responders, the median (IQR) SARS-CoV-2 anti-spike antibody titer was 666.7 (578.9–880.6) 5 months after a second dose of the BNT162b2 vaccine (Figure 1c).

SARS-CoV-2 anti-spike antibody was produced by both suboptimal and normal responders, but levels were lower at 5 months than at 1 week after a second dose of the vaccine (Figure 1).

### 3.2. SARS-CoV-2 Neutralizing Antibody Activity in Participants with Suboptimal Antibody Responses to Previous Antiviral Vaccines

**Figure 2:**
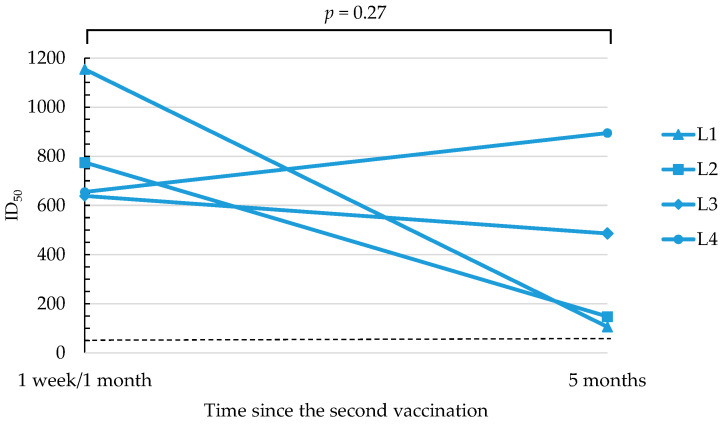
SARS-CoV-2 neutralizing antibody activity responses after the second dose of BNT162b2 vaccine in suboptimal responders. Data are presented as 50% inhibitory dilutions (ID_50_). The dashed line indicates the cutoff for ID_50_ [20].

SARS-CoV-2 neutralizing antibody titers after vaccination in suboptimal responders were assessed 1 week after a second dose of the BNT162b2 vaccine; however, since sample volumes for participants L2, L3, and L4 were insufficient at 1 week, their titers were re-evaluated at 1 month. Participant L1 had a SARS-CoV-2 neutralizing antibody titer of 1154.0 1 week after receiving a second dose of the BNT162b2 vaccine; participants L2, L3, and L4 had titers of 774.2, 638.3, and 654.6, respectively, 1 month after receiving a second dose (Figure 2). Five months after the second vaccination, SARS-CoV-2 neutralizing antibody titers were 105.5, 147.7, 485.2, and 894.4 for L1, L2, L3, and L4, respectively (Figure 2). All four suboptimal responders showed neutralizing antibody activity within the protective range (≥50), at 1 week/1 month as well as at 5 months after the second vaccination (Figure 2). SARS-CoV-2 neutralizing antibody levels in suboptimal responders were generally lower at 5 months after the second dose of the vaccine compared to the levels at 1 week/1 month, but this difference was not statistically significant (*p* = 0.27) (Figure 2).

## 4. Discussion

The BNT162b2 vaccine has been highly effective in preventing clinically significant COVID-19 [8,9,10,11]. However, there is limited information available about the effectiveness of the BNT162b2 vaccine in individuals with a suboptimal response to conventional vaccines. In this study, suboptimal responders produced adequate SARS-CoV-2 anti-spike antibodies and neutralizing antibodies.

Muik et al. [22] measured the neutralization efficacy of sera from 40 healthy German individuals immunized with the BNT162b2 vaccine and a 50% inhibitory dilution factor using pseudovirus according to the same method that was used in the current study. Among the suboptimal responders in the current study, the SARS-CoV-2 neutralizing activity 1 week/1 month after receiving a second dose of the BNT162b2 vaccine was comparable to that of the participants in the German study. All four suboptimal responders developed protective levels of neutralizing antibodies. SARS-CoV-2 neutralizing antibody activity 5 months after a second dose of the BNT162b2 vaccine did not show a significant decrease in activity compared to activity at 1 week/1 month after the second dose. In the suboptimal responders in the current study, SARS-CoV-2 anti-spike antibody titers were generally lower at 5 months after a second dose of vaccine than at 1 week, but the decrease was not statistically significant. These results suggest that mRNA vaccines can invoke a protective immune response even in individuals with a suboptimal response to conventional vaccines against viral diseases such as mumps and measles.

Measles, rubella, chickenpox, and mumps vaccines are live attenuated vaccines that weaken the toxicity of pathogenic microorganisms, and hepatitis B vaccines are inactivated vaccines that cause pathogenic microorganisms to lose their ability to infect [23]. In previous studies, approximately 2–10% of healthy individuals did not produce protective antibody levels in response to viral vaccines [24]. The risk factors for suboptimal responses to conventional viral vaccines include male sex, age of >40 years, smoking, obesity, chronic diseases, and genetic factors. However, the factors associated with a low antibody response to SARS-CoV-2 mRNA vaccines are not well understood. Smokers, patients with cancer and hematologic malignancies, and organ transplant recipients have been reported to have a lower SARS-CoV-2 anti-spike antibody response than individuals without these characteristics after receiving SARS-CoV-2 vaccines [25,26,27,28,29,30]. It is noteworthy that the suboptimal responders in the current study who did not produce adequate antibodies against measles or mumps vaccines produced adequate SARS-CoV-2 anti-spike antibodies after receiving the BNT162b2 vaccine. One possible reason for this discrepancy is the different mechanisms underlying antibody production for conventional vaccines and for SARS-CoV-2 mRNA vaccines.

The BNT162b2 vaccine is an mRNA vaccine that incorporates mRNA encoding spike protein (antigen) from the surface of the SARS-CoV-2 virus into human cells [31]. The produced antigen directly functions as a template for the acquired immune system [32]. On the other hand, live attenuated vaccines or inactivated vaccines need to be processed so that they can be recognized as antigens. Differences in underlying action mechanisms could be a factor that contributed to the discrepancy in responses to the BNT162b2 vaccine and conventional live attenuated vaccines among our participants, with a suboptimal response to conventional vaccines. This hypothesis should be tested in future studies. It is unclear why the suboptimal responders in the current study had higher antibody titers than normal responders in the first week after a second dose of the BNT162b2 vaccine.

SARS-CoV-2 anti-spike antibody levels are known to peak and then decrease within months after the second vaccination, which represents a much shorter period than that observed for antibodies against live attenuated vaccines [33,34]. In order to achieve adequate COVID-19 disease control, a third dose of SARS-CoV-2 mRNA vaccine is therefore needed and is already being administered in many countries. In view of the results of the current study, we recommend that a third dose of the BNT162b2 vaccine be administered to HCWs regardless of whether they have shown a suboptimal or normal response to conventional vaccines in the past [33].

Our study has some limitations. Only four participants in our sample were suboptimal responders, and all participants were HCWs working and vaccinated at the same hospital. In addition, no suboptimal responders to inactivated vaccines (such as hepatitis B) were included in this study sample. Multicenter studies with larger sample sizes are therefore needed to confirm the current results. In addition, further immunological studies are necessary to clarify the mechanism by which humoral immunity is acquired after mRNA vaccination in suboptimal responders to conventional vaccines.

In conclusion, after two doses of the BNT162b2 vaccine, suboptimal responders to conventional antiviral vaccines developed protective levels of SARS-CoV-2 anti-spike and SARS-CoV-2 neutralizing antibodies. These results suggest that SARS-CoV-2 mRNA vaccines should be administered to individuals with a suboptimal response to conventional vaccines as well as to normal responders.

## Data Availability

Not applicable.
